# Encyclopædia Medica

**Published:** 1900-06

**Authors:** 


**Encyclopædia Medica.** Under the General Editorship of CHALMERS WATSON, M.B. Vol. III.—*Diphtheria* to *Food.* Pp. vi., 545. Edinburgh: William Green & Sons. 1900.

The principal articles of this volume number forty-five, the work of as many different writers. The first, on *Diphtheria*, is by Dr. E. W. Goodall, a great authority on this subject, and his views are very definite. "The cardinal rule in the antitoxic [treatment] is—Inject early. If the treatment is commenced on the first day the dose should be 1500 units at least; it will usually be unnecessary to give more than 2000. But if it be delayed, the amount must be increased up to 8000 or 10,000 units, according to the severity of the case. It is advisable to repeat from half to the whole first dose within twenty-four hours if the local exudation shows no sign of resolution. With respect to the total amount to be administered, though as far as the writer knows (and he has often injected from 30,000 to 50,000 units) the limit is set only by the volume of the serum that can with convenience be injected, yet his experience leads him to say that little is to be gained by giving more than 16,000 units during the first twenty-four hours from the commencement of the treatment." He approves of local treatment by mild alkaline boric douches, and for private practice he prefers tracheotomy to intubation, which latter procedure he considers to be inadmissible and inexpedient in a considerable proportion of the cases. We cannot endorse the rather sweeping condemnation of intubation in private practice, for it is only in exceptional circumstances that skilled assistance cannot be available for all difficulties that are likely to arise. The author seems to be unacquainted with O'Dwyer's special tubes for loose membrane, through which membranes may be readily expectorated. He advises that the patient should be kept in isolation for at least four weeks from the commencement of the illness. His statistics of the mortality in the Metropolitan Asylums Board Hospitals show a reduction in the case of those admitted on the first day of the illness from 22.5 to 3.8 per cent.; on the second day, from 27.0 to 12.1; and on the third day, from 29.4 to 20.9; while among those admitted later there has been little improvement.

Dr. Willoughby gives excellent rules for *disinfection*, and considers that the common method of aerial fumigation by means of sulphur is very inadequate. He advises a solution of formaline, 1 part to 20 of water, for clothing.

A chapter on *Drug Eruptions*, by Glasgow Pattison, includes an account of the antitoxin rashes: he observes that the antitoxic principle employed has nothing to do with their causation, nor are they due to any antiseptic which may be used in their preservation. They depend entirely on the serum itself, as is proved by the fact that the normal horse serum produces them, and are in some way connected with the constitution of the individual horse.

No one is better suited than Dr. Davidson to describe the details of *Dysentery*, whose study leads to the conclusion that the active and essential pathological entity is a variety of the bacillus coli, and other than the amœbæ which have been demonstrated in some varieties of the disease.

Seven papers on *Diseases of the Ear*, all by well-known specialists occupy ninety pages, and leave little further to be said thereon.

The article on *Eczema* by Dr. Leslie Roberts is very original, and its treatment resolves itself into a definite piece of work in dynamics. All successful treatment is based on local expurgation, and must be directed to the assisting and accelerating of this process. He attaches no importance to any constitutional treatment, and states that the importance of diet is commonly exaggerated.

Glénard's disease, *Enteroptosis*, is described by Dr. Gillespie; *Epilepsy and Epileptic Colonies* by Dr. Aldren Turner, who is visiting physician to the colony at Chalfont; *Facial Hemiatrophy, Paralysis, and Spasm* by Dr. J. S. Risien Russell. Many other articles are worthy of especial mention, and a chapter on *Food*, by Dr. E. W. Hope, points out the differences between milk as Nature intended it and milk which has gone through the many vicissitudes which necessitate its sterilisation. This concludes a volume styled by a weekly journal as "The book of the month."

